# Frequency of thyroid incidentalomas in Karachi population

**DOI:** 10.12669/pjms.304.4808

**Published:** 2014

**Authors:** Mahrukh Kamran, Nuzhat Hassan, Muhammad Ali, Farah Ahmad, Sikandar Shahzad, Nosheen Zehra

**Affiliations:** 1 Dr. Mahrukh Kamran, MBBS, Senior Lecturer, Department of Anatomy, Ziauddin College of Medicine, Ziauddin University, Karachi, Pakistan.; 2 Nuzhat Hassan, Masters in Philosophy (M. Phil), Anatomy, Professor and Chairperson Department of Anatomy, Ziauddin University, Karachi, Pakistan.; 3 Dr. Muhammad Ali, FCPS, Fellowship in VIR, Fellowship in VIR, Assistant Professor, Department of Radiology, Head Clifton Campus, Ziauddin University Hospital, Karachi, Pakistan.; 4 Dr. Farah Ahmad, Assistant Professor C.H.S Department, Ziauddin University Hospital, Karachi, Pakistan.; 5 Dr. Sikander Shahzad, MD.Resident Radiology Department, Ziauddin University Hospital, Karachi, Pakistan.; 6 Dr. Nosheen Zehra, Assistant Professor C.H.S Department, Ziauddin University Hospital, Karachi, Pakistan.

**Keywords:** Thyroid Gland, Thyroid Nodule, Ultrasonography, Thyroid Hormones, Thyroid Function Tests, Thyroid Neoplasms

## Abstract

***Objectives:*** The aim of this study was to determine frequency of thyroid incidentalomas (TI) through ultrasound (US) and its association with age, gender and ethnicities.

***Methods:*** It was a cross-sectional study. Total 269 adults who were asymptomatic for thyroid disease aged 21 years and above underwent ultrasound examination of their thyroid.

***Results:*** Frequency of TI found was 21%. TI was detected in 25% of females and 16% males (P= 0.078). 61% had thyroid nodules (TNs) in one lobe (right, left or isthmus) and 39% had TNs in more than one location. About 55% had single TN and 45% had multiple TNs. 38% had TNs greater than 1cm while 57% had TNs smaller than 1 cm. 5% had TNs greater and smaller than 1 cm. TI was equally common in individuals of different ethinicities (P= 0.758).

***Conclusion:*** Frequency of thyroid incidentalomas found in our study was higher than most of the other iodine sufficient states. Unlike other studies, incidentalomas were equally common in both the genders of our study. This may be due to the previous iodine deficient status of Pakistan which was prevalent. However further studies on the same topic will help us in identifying the correct status of thyroid incidentalomas if Pakistan retains it’s status as an iodine sufficient state.

## INTRODUCTION

Advancement in medical technology has increased the frequency of detection of subclinical nodules in endocrine organs especially in thyroid gland called “Thyroid incidentalomas” (TI). TI means discovery of a thyroid nodule (TN) by imaging investigations in individuals who are asymptomatic for thyroid disease.^1^^,^^2^ A TN is simply an atypical growth of follicular cells of thyroid, which form swelling in the gland. A thyroid swelling is palpable when it is large or when it is present close to the surface of gland. TN is called functioning when it is secreting thyroid hormones and it is called non-functioning when not secreting thyroid hormones. TN can be single or multiple, solid or cystic and can be benign or malignant.^3^^,^^4^

TNs are generally asymptomatic with normal thyroid function tests. Many causative factors have been identified; Among these adenomas, cysts and thyroiditis, predominantly Hashimoto’s thyroiditis are the regular risk factors for increased frequency of TI.^3^^,^^4^ Previous studies have revealed that frequency also increases with increasing age and it is more common in females. These nodules are also more prevalent in areas of endemic goiter like Sudan, Ethopia, Ghana and Morocco and high exposure to radiation.^4^^,^^5^^-^^8^^,^^9^

Frequency of TI is greatly influenced by the method of detection. According to the study by Wiest et al. only 6.4% of TN size less than 0.5cm can be detected by palpation.^10^ Overall frequency of TN detection by palpation is 2% to 21%. While ultrasound (US) can detect TN as small as 2mm reaching the frequency of 19% to 67% which was comparable to detection of TI at postmortem examination (30% to 60%).^1^^,^^5^^-^^8^^,^^10^^,^

During the palpation of thyroid gland, one can miss small or deeply situated nodule. This method is also not reliable in determining the type of nodule (solid, cystic or mixed) and in identifying all the nodules in case of multiple nodules.^6^^,^^10^

US is a non invasive, rapid, cost-effective, safe and accurate method by which one can detect small and deep TNs that are missed by palpatory method. This method can detect size, shape as well as some malignant characteristics of nodules. Furthermore, it can determine regional lymphadenopathy in cases of malignancy and due to its visual surveillance, it can also be used for therapeutic purposes. A TN that is discovered during US examinations has an equal potential to be malignant as a palpable TN.^10^^-^^13^ US characteristics of malignant TNs are entirely hypoechoeic solid masses with micro-calcifications and increased blood flow in the center.^10^^-^^14^

If a TN produces excess of thyroid hormone it is called a hot or toxic nodule. This leads to hyperthyroidism and most commonly thyrotoxicosis which is detected by thyroid function test.^3^ Moreover there is always a fear of nodule turning malignant. This requires early detection and prompt management of TN. Studies carried out previously have revealed that between 0.45% to 13% of thyroid incidentalomas have potential to become malignant.^6^ This frequency reaches upto 33% when detected by PET- scan.^3^

Like rest of the world, benign TNs are also more common in Pakistan when compared to malignant TNs. Frequency of benign TNs reported in Pakistan is 89%. Among the benign lesion adenomatous goiter is the most prevalent reaching the frequency of 68%.^15^ Frequency of thyroid malignancy in Pakistan ranges from is 11%-14.35%.^15^^,^^16^ Zuberi LM et al reported that in Pakistan thyroid cancer is more aggressive and common in 30 to 60 years of age.^16^ Thyroid cancer is twice as common in females as compare to males. Female to male ratio of thyroid cancer reported in Pakistan is 2.2:1.^16^ The main types of thyroid cancers found here are papillary, follicular and anaplastic carcinomas. Papillary carcinomas had the highest frequency of 73.7%, followed by follicular and anaplastic carcinomas with the frequency of 20% and 6.7% respectively.^15^

In this study we have tried to determine the frequency of TI through US in adults coming for investigation of conditions other than thyroid disease in a tertiary care hospital in Karachi and also to determine its association with age, gender and ethnicity.

## METHODS

A cross-sectional study was carried out over a period of 6 months from November 2012 to April 2013. Total 269 volunteers age 21 years and above participated in the study through convenience sampling. After thyroid palpation the participants underwent US examination at Radiology Department of Ziauddin Hospital.

Sample was calculated by applying formula: n= Z^2^ x P (1 –P)/d^2^. Where

n= no of individuals

P= 13.6% (TI in Iran)^17^

Z =standard error of mean= 1.96

d =absolute precision 5% = 0.05 

All individuals with history of thyroid disease or with thyroid surgery were excluded from the study. Individuals with palpable TN were also excluded. Written informed consent was taken and proforma was filled.

Ultrasound (US) machine Toshiba SSA-590A with a 7.5 MHz linear probe was used to evaluate thyroid gland. Volunteers were examined in supine position with their neck hyper extended and pillow under their shoulders. Antero-posterior, cranio-caudal, medio-lateral views of both lobes of thyroid were taken into account as well as isthmus thickness was also noted. The study was approved by ethical review committee of Ziauddin University Karachi.

***Statistical Analysis: ***Statistical analysis was carried out on SPSS version 17. Chi- square was applied to determine the association of TN with age, gender and ethnicity. Independent student’s T-test was applied to compare the means of age with nodule and without nodule. P- Value of 0.05 or less was considered to be significant.

## RESULTS

Out of 269 individuals 140 were females (52%) and 129 were males (48%). There was a significant difference in the mean age of the individuals with nodules 48.8 ± 14.8 years and without nodules 39.25 ± 14.8 years (P=0.00). Overall frequency of TI was 21% (56 out of 269). TI was insignificantly frequent in females 25% (35 out of 140) as compare to males 16% (21 out of 129) (P= 0.078) (Table-I).

**Graph.1:**
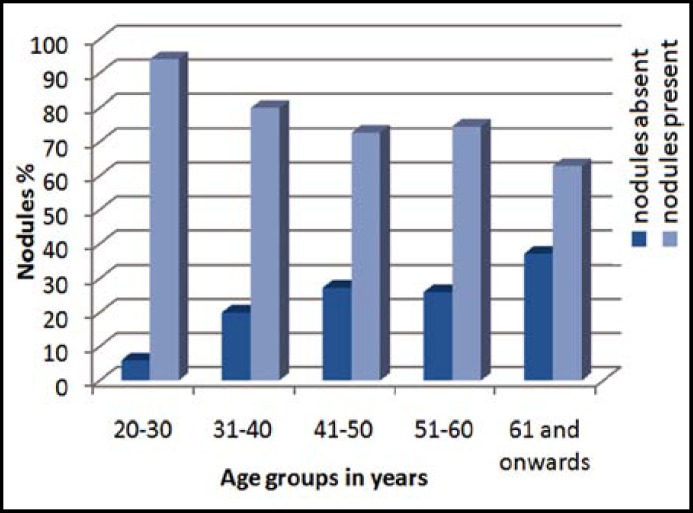
Percentage of individuals with and without nodules.

In our study frequency of TNs increased with age (Graph.1) and there was a significant difference in TI among the different age groups (P= 0.002) (Table-I). However, TI was equally common among individuals of all ethnicities (P=0.758) (Table-I).

**Graph.2:**
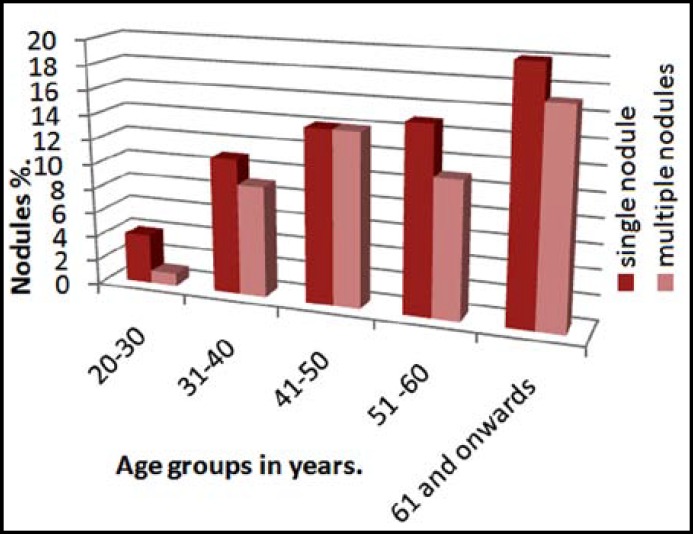
Percentage of single and multiple of nodule(s) according to age

About 55% (31 out of 56) individuals in the study had single nodule and 45% (25 out of 56) had multiple nodules. Percentage of single nodule and multiple nodules of thyroid gland in different age groups is given in Graph.2.

About 39% (22 out of 56) individuals had TNs in more than one location. 38% (21out of 56) individuals had TNs only in right lobe, 21% (12 out of 56) individuals had TNs only in left lobe and 2% (1 out 56) individuals had TNs only in isthmus.

About 38% (21 out of 56) individuals in the study had TNs greater than 1cm and 57% (32 out of 56) individuals had TNs smaller than 1cm. 5% individuals (3 out of 56) had TNs greater and smaller than 1 cm.

## DISCUSSION

Examination of thyroid gland by palpation has been the most common method for detection of TNs. However, it overlooks small and deeply situated nodules. Remarkable innovation in medical technology has led to considerable increase in the detection of TNs. Thyroid Ultrasound when compared to other modalities is a more accurate, non invasive and economical. Characteristics of nodule like echogenicity (hypoechoic or hyperechoic), composition (solid, cystic and mixed), presence or absence of calcification, margins (regular or irregular) and blood flow ( increased or decreased, central or peripheral) can also be identified by US.^10^^-^^13^ Wiest et al. in 1998 suggested that examination through high resolution US guarantees great sensitivity, high specificity and good scanning speed.^10^

Frequency of TI varies in different regions of the world. Many factors have been reported for these variations of which most common are age, gender, and iodine intake.^5^^,^^7^^,^^8^ Frequency of TI in Iran is 13.6%,^17^ in U.S.A is 9.4%^18^ and is 13.4% in California,^19^ in Korea is 36.67%,^20^ in Poland is 14.8%^21^ and in Finland is 27.35%.^7^ Frequency of TI found in our study was slightly greater than most of iodine sufficient area. This could be due to recent evolution of Pakistan as an iodine sufficient state.^9^

In our study mean age of the individuals with TI was 48 ± 14.8 years and without TN was 39.25 ± 14.8 years which is in contrast to the study by Taheri et al in Iran.^22^ In this study mean age of people with nodule and without nodule was 63.3 ± 12.9 years and 61.7 ± 13.6 years respectively.

Frequency of TI increases with age^7^^,^^10^^,^^12^^-^^14^^,^^17^^&^^18^ which was observed in our study as well (Graph.1) (Table-I). However frequency of TI after 60 years of age (37.1%) was little lower in our study when compared with that of American Thyroid Association (50%).^4^

**Table-I:** Characteristics of individuals with nodule and without nodule

		***Nodule + ve***		***Nodule –ve***		***P value***
		***N***	***%***	***N***	***%***	
Gender	Male	21	16.3	108	83.7	0.078
	Female	35	25	105	75	
Age	20-30	4	5.9	64	94.1	0.002**
	31-40	15	20	60	80	
	41-50	12	27.3	32	72.7	
	51-60	12	25.5	35	74.5	
	60 onwards	13	37.1	22	62.9	
Ethnicity	Urdu speaking	18	21.7	65	78.3	0.758
	Sindhi	8	30.8	18	69.2	
	Punjabi	9	21.4	33	78.6	
	Pathan	19	21.1	71	78.9	

** P- Value of 0.05 or less was significant.

Previous studies has suggested that frequency of TI is greater in females.^7^^,^^8^^,^^13^^,^^17^^,^^21^^,^^22^ Barbara et al. in 1982 reported 7.9% of males and 20.6% of female in USA had asymptomatic TNs.^19^ In our study frequency of TNs was higher in females (25%) when compared to males (16%) but these findings were insignificant statistically (P=0.078). As Pakistan was a severe iodine deficient state we suggest that iodine deficiency in recent past could be the primary cause for increased frequency of TI in both the genders.^23^

Taheri et al. in 2008 reported that 51.9% of Iranian population had TN in one lobe (right, left or isthmus) and 48% individuals had TN in more than one locations.^22^ However, Steel et al reported 49.4% individuals with bilateral TNs and 50.6% individuals with unilateral TNs.^18^ Results of our study were similar to both the studies but unilateral nodules (60.7%) were much more prevalent than bilateral nodules (39%).

It is important to classify TN as single nodule and multiple nodules as Zuberi LM in 2004 reported that in Pakistan risk of thyroid malignancy was more in multiple nodules.^16^ Difference in frequency of single nodule (55%) and multiple nodules(45%) in our study was much smaller as compared to the studies by Taheri et al. and Brander et al. Taheri et al. reported that single nodule was more common in Iranian population as 61% individuals had single nodule and 39% had multiple nodules.^22^ Study by Brander et al. from Finland had supported Taheri et al. and prevalence of single and multiple nodules in their population was 57% and 22% respectively.^7^

Risk of developing malignancy in TN is equal in nodule greater and smaller than 1 cm in size^14^ and US of thyroid for nodular thyroid diseases offers great sensitivity and high specificity.^10^ In our study frequency of nodule smaller than 1 cm was 57% and nodule greater than 1 cm was 38% and only 5% individuals had nodules smaller and greater than 1 cm. These findings were close to frequencies given by Mohammadi et al. where 61.8% individuals had nodule smaller than 1cm, 33.9% individuals had nodule larger than 1 cm and 6.3% individuals had nodules greater and smaller than 1 cm.^17^ However Brander et al. in his study reported frequency of 70% for nodule smaller than 1cm.^7^

In our study TN were equally common in individuals of different ethnicities (Table-I). There are probably two reasons for this. Firstly, in Muslims there are fewer religious restrictions on intake of seafood.^24^ Secondly there is an easy access to sea food leading to increase in its consumption in our population due to coastal location. 

***Limitations of this study: ***Only frequency of TI was determined in our population. Prevalence was not determined as our study was limited to the individuals coming to Ziauddin Hospital Clifton. Secondly use of 3-dimensional US would have been more accurate as it is automated and is reproducible when compared with 2–dimensional US.^25^^&^^26^ Although the study showed that TN was common in all the ethnicities but we cannot generalize the finding because of small sample size.

## CONCLUSION

Frequency of thyroid incidentalomas found in our study was higher than most of the other iodine sufficient states. Unlike other studies, incidentalomas were equally common in both the genders of our study. This may be due to the previous iodine deficient status of Pakistan which was prevalent. However further studies on the same topic will help us in identifying the correct status of thyroid incidentalomas if Pakistan retains it’s status as an iodine sufficient state.

## Authors Contribution:

MK: Selected the study topic drafting the synopsis, collecting and analyzing the data and drafting the final manuscript.

NH: Critical analysis of the study.

MA: Final approval and analyzing the study clinically.

FA: Support in statistics and methodology and critically analyzing it

SS: For technical support and clinical analysis. 

NZ: For critical analysis.

## Notes

***Funding resources:*** Ziauddin University and Self finance.
